# Uptake of the *Fusarium* Effector Avr2 by Tomato Is Not a Cell Autonomous Event

**DOI:** 10.3389/fpls.2016.01915

**Published:** 2016-12-21

**Authors:** Xiaotang Di, Jo Gomila, Lisong Ma, Harrold A. van den Burg, Frank L. W. Takken

**Affiliations:** Molecular Plant Pathology, Faculty of Science, Swammerdam Institute for Life Sciences, University of AmsterdamAmsterdam, Netherlands

**Keywords:** effector uptake, *Fusarium oxysporum*, *Verticillium dahliae*, tomato, grafting, *Agrobacterium tumefaciens*

## Abstract

Pathogens secrete effector proteins to manipulate the host for their own proliferation. Currently it is unclear whether the uptake of effector proteins from extracellular spaces is a host autonomous process. We study this process using the Avr2 effector protein from *Fusarium oxysporum* f. sp. *lycopersici* (*Fol*). Avr2 is an important virulence factor that is secreted into the xylem sap of tomato following infection. Besides that, it is also an avirulence factor triggering immune responses in plants carrying the *I-2* resistance gene. Recognition of Avr2 by I-2 occurs inside the plant nucleus. Here, we show that pathogenicity of an *Avr2* knockout *Fusarium* (*FolΔAvr2*) strain is fully complemented on transgenic tomato lines that express either a secreted (Avr2) or cytosolic Avr2 (ΔspAvr2) protein, indicating that Avr2 exerts its virulence functions inside the host cells. Furthermore, our data imply that secreted Avr2 is taken up from the extracellular spaces in the presence of the fungus. Grafting studies were performed in which scions of *I-2* tomato plants were grafted onto either a *ΔspAvr2* or on an *Avr2* rootstock. Although the Avr2 protein could readily be detected in the xylem sap of the grafted plant tissues, no I-2-mediated immune responses were induced suggesting that *I-2-*expressing tomato cells cannot autonomously take up the effector protein from the xylem sap. Additionally, *ΔspAvr2* and *Avr2* plants were crossed with *I-2* plants. Whereas *ΔspAvr2/I-2* F1 plants showed a constitutive immune response, immunity was not triggered in the *Avr2/I-2* plants confirming that Avr2 is not autonomously taken up from the extracellular spaces to trigger I-2. Intriguingly, infiltration of *Agrobacterium tumefaciens* in leaves of *Avr2/I-2* plants triggered I-2 mediated cell death, which indicates that *Agrobacterium* triggers effector uptake. To test whether, besides *Fol*, effector uptake could also be induced by other fungal pathogens the *ΔspAvr2* and *Avr2* transgenic lines were inoculated with *Verticillium dahliae.* Whereas *ΔspAvr2* plants became hyper-susceptible to infection, no difference in disease development was found in the *Avr2* plants as compared to wild-type plants. These data suggest that effector uptake is not a host autonomous process and that *Fol* and *A. tumefaciens*, but not *V. dahliae*, facilitate Avr2 uptake by tomato cells from extracellular spaces.

## Introduction

Microbe-secreted effector proteins enable pathogens to suppress or evade plant immunity responses, a prerequisite for successful infections. Most bacterial plant pathogens employ a type-III secretion system to directly inject type-III effector (T3E) proteins into the plant cytoplasm (Panstruga and Dodds, 2009). Fungi and oomycetes do not inject their effectors into plant cells, but secrete them into the extracellular spaces. Some fungal pathogens, such as *Cladosporium fulvum*, secrete effectors from invasive hyphae into the plant apoplast (Stergiopoulos and de Wit, 2016). Others, like *Magnaporthe grisea* and *Phytophthora infestans*, have specialized feeding structures protruding into the plant cells from which effectors are secreted (Panstruga and Dodds, 2009; Dodds and Rathjen, 2010). In either case, the effectors accumulate outside the host’s plasma membrane and it is unknown how they are taken up by plant cells (Bozkurt et al., 2012; Rafiqi et al., 2012). As many effectors have been shown to act inside the host cell (Dodds et al., 2004; Armstrong et al., 2005; Catanzariti et al., 2006), there must be a mechanism by which effector proteins enter. Whether this process is a host autonomous mechanism or requires the presence of the pathogen is currently an unresolved question. To address this question its desirable to use a pathosystem in which effector secretion and action are spatially separated. In this study we focus on the fungal pathogen *Fusarium oxysporum*, which secretes its effector proteins into the xylem sap of infected plants (Houterman et al., 2007). The effectors are transported with the sap stream to exert their action in various places in the plant.

*Fusarium oxysporum* is a soil-borne and highly destructive pathogen causing vascular wilt disease on a wide range of plants. The *F. oxysporum* species complex comprises different *formae speciales* (*f. sp*.), which collectively infect more than 100 different hosts, provoking severe losses in crops such as melon, tomato, cotton and banana, among others (Michielse and Rep, 2009). The process of infection by *F. oxysporum* can be divided into several steps: root recognition, root surface attachment and penetration, colonization of the root cortex and, in the case of wilt-inducing *formae speciales*, hyphal proliferation within the xylem vessels (Pietro et al., 2003). Characteristic disease symptoms include vascular browning, leaf epinasty, stunting, progressive wilting, defoliation and eventually plant death.

In the past decades, the interaction between tomato and *F. oxysporum* f. sp. *lycopersici* (*Fol*) has evolved into an excellent model to study the molecular mechanisms underlying disease and resistance (Takken and Rep, 2010). Over 14 putative effector proteins have been isolated from the xylem sap of infected tomato plants and are called Six (for secreted in xylem) proteins (Houterman et al., 2007). For some of them, like Six1, Six3, Six5 and Six6 a virulence function has been determined, making them effectors *in sensu stricto* (Rep et al., 2005; Houterman et al., 2009; Gawehns et al., 2014; Ma et al., 2015). Besides a virulence function some effectors have been found to act as avirulence determinants, triggering immune responses in resistant hosts. The relationship between *Fol* and tomato cultivars follows the ‘gene-for-gene’ hypothesis (Flor, 1971). According to this hypothesis disease resistance conferred by resistance (R) genes requires ‘matching’ avirulence (Avr) genes in the pathogen. Three *R* genes against *Fol* have been introgressed into cultivated tomato (*Solanum lycopersicum*): the *I* and *I-2* genes from *S. pimpinellifolium*, which confer resistance against *Fol* races 1 and 2, respectively, and the *I-3* gene from *S. pennellii*, which confers resistance to *Fol* race 3. The three *Fol* effector proteins Avr1 (Six4), Avr2 (Six3) and Avr3 (Six4), which are recognized by *I, I-2* and *I-3*, respectively, have all been cloned (Rep et al., 2004; Houterman et al., 2008, 2009). They are secreted into the xylem sap during infection. Avr3 is expressed when the fungus is in contact with living plant cells (van der Does et al., 2008) while Avr2 is predominantly expressed in xylem-colonizing hyphae (Ma et al., 2013). Both Avr3 and Avr2 are important for pathogenicity (Rep et al., 2005; Houterman et al., 2009). Notably, Avr1 does not enhance virulence on a susceptible plant, but suppresses *I-2* and *I-3*-mediated resistance allowing the fungus to overcome the gene-for-gene resistance (Houterman et al., 2008).

*Avr2* encodes a mature 15.7 kDa protein preceded by an N-terminal signal peptide. Avr2 contains two cysteine residues that might form a disulfide bond (Houterman et al., 2007). The protein appears in various positions in 2D gels of xylem sap from *Fol*-infected tomato plants, corresponding to apparent sizes from 11 to 14 kDa, probably as a result of proteolytic processing from the N-terminus (Houterman et al., 2007). Race 3 strains carry point mutations in *Avr2*, resulting in single amino acid changes that do not affect its virulence function but allow the protein to evade *I-2*-mediated recognition (Houterman et al., 2009). Although Avr2 is secreted into xylem sap, the Avr2 protein is recognized intracellularly in the plant nucleus by I-2 (Ma et al., 2013), implying uptake by host cells. Here, we describe the generation of transgenic tomato plants expressing either full-length *Avr2* or a truncated version lacking the signal peptide encoding sequence (*ΔspAvr2*). Bioassays and grafting studies using these plants revealed that Avr2, besides it avirulence function, also exerts its virulence function inside host cells. Having an extracellular effector that is secreted in the xylem sap, but exerts its functions inside the host cell, makes this a perfect model to study effector uptake and to reveal whether uptake is a host autonomous process or requires the presence of the pathogen.

## Materials and Methods

### Plant Material and Fungal and Bacterial Strains

Tomato (*Solanum lycopersicum*) cultivar Moneymaker, which is susceptible to *Fol* race 2 *Fol007*, and a resistant cultivar, 90E341F, which contains the *I-2* resistance locus were used (Stall and Walter, 1965; Kroon and Elgersma, 1993). Tomato plants were germinated and grown on soil with 16/8 h light/dark cycles, at 22/16°C day/night and 70% relative humidity in a temperature-controlled green house. *FolΔAvr2* carrying a deletion of the *Avr2* gene in the *Fol007* background was described previously (Houterman et al., 2009).

### Construction of Binary Vectors

Full length *Avr2* was PCR-amplified with primers FP2524 (5′-CGCTCTAGAATGCGTTTCCTTCTGCTTAT-3′) and FP2274 (5′-GCGGGATCCTCCATCCTCTGAGATAGTAAG-3′) using CTAPi::*Avr2* as template (Houterman et al., 2009). The obtained products were cloned into the vector pSLDB3104 (Tameling et al., 2010) between the *Xba*I and *Bam*HI restriction sites to generate SLDB3104::*Avr2*. SLDB3104::*ΔspAvr2* has been described before (Ma et al., 2013). All PCR primers were purchased from MWG^1^ and sequences of all plasmids were confirmed by sequence analysis. *Avr2* and *ΔspAvr2* were cloned behind the cauliflower mosaic virus 35S promoter for constitutive expression and fused to a C-terminal hemagglutinin (HA) and streptavidin-binding peptide (SBP) tag. The resulting vector was introduced by electroporation into LBA4404 (Hoekema et al., 1983) for tomato transformation.

### Plant Transformation

Moneymaker was transformed with the construct described above using *Agrobacterium*-mediated transformation as described before (Cortina and Culianez-Macia, 2004). Briefly, surface-sterilized seeds were sown on Murashige and Skoog (MS) agar supplemented with sucrose (15 g/l). The seeds were incubated in the dark in a growth chamber at 25°C for 2 days, and subsequently exposed to light. After 10 days, the base and the tip of the cotyledons was removed and the cotyledons were placed upside up in Petri dishes containing co-cultivation medium (MS agar supplemented with 30 g/l sucrose, 0.5 g/l 2-(*N*-orpholino) ethanesulfonic acid (MES) [Duchefa] and 0.2 mM Acetosyringone, pH 5.75). The plates were incubated for 24 h at 25°C in dark. Transgenic *Agrobacterium tumefaciens* carrying the construct of interest was grown in 30 ml LBman at 28°C overnight (maximum 16–18 h). After harvesting, the bacteria were resuspended in 30 ml LM2 medium (4.4 g/l MS, 30 g/l sucrose, 0.5 g/l MES [Duchefa] and 0.2 mM Acetosyringone, pH 5.75). Subsequently, the explants were incubated in the bacterial suspension for maximal 1 min, briefly dried on sterile filter paper and placed on co-cultivation plates. The plates were incubated in the dark for 48 h at 25°C after which the explants were transferred to selection plates (MS agar supplemented with 30 g/l sucrose, 0.5 g/l MES, 0.5 mg/l zeatin riboside, 0.5 mg/l indole-3-acetic acid (IAA), 250 mg/l carbenicilline, 100 mg/l vancomycin, and 40 mg/l kanamycin, pH 5.75). Explants were transferred to fresh selection plate every 2 weeks. When callus appeared, it was transferred to new selection plates until shoots appeared. Upon shoot development, the shoots were harvested and transferred to root-inducing medium (MS agar supplemented with 15 g/l sucrose, 0.5 g/l MES, 4 g/l gelrite, 50 mg/l kanamycin, pH 5.75). Once roots developed, the plantlets were potted in soil and transferred to the greenhouse where they were grown under standard greenhouse with conditions of a 16 h photoperiod and 70% relative humidity at 25°C.

First-generation transformants of *ΔspAvr2* and *Avr2* were selected on 1/2 MS medium containing kanamycin (40 mg/l). For the *ΔspAvr2* transgenic line, 25 seeds of nine T1 progeny were analyzed for segregation by scoring the ratio of kanamycin-resistant to kanamycin-sensitive seedlings. Six lines segregated roughly 3:1 for green versus yellowing seedlings. Subsequently the kanamycin-resistant plants were transferred to soil and self-fertilized. Homozygous single insertion lines were selected from the kanamycin resistant T2 plants according to their segregation pattern. Of each independent T2 line 25 plants were checked by PCR with primer pairs FP962 (5′-TGAGCGGGCTGGCAATTC-3′) and FP963 (5′-CAATCCTCTGAGATAGTAAG-3′) detecting a 273-bp fragment of the *Avr2* gene. Two lines were homozygous for the *Avr2* transgene (*ΔspAvr2-3 and ΔspAvr-30*). Homozygous *Avr2* transgenic lines were screened using the same procedure. Eventually three of 23 *Avr2* plants (*Avr2-1, Avr2-4*, and *Avr2-7*) were kept for further study.

Primer pairs FP962 and FP963, and FP484 (AAAGCGTGGTATTGCGTTTC) and FP165 (TTCCGGATGTCCCATAGGATCC) were used to amplify *Avr2* and *I-2* from genomic DNA of *Avr2/I-2* and *ΔspAvr2/I-2* plants, respectively.

### Protein Extraction and Western Blotting

Protein extraction was done as described previously (Ma et al., 2015). To verify presence of Avr2 in transgenic tomato plants, leaves were harvested and snap-frozen in liquid nitrogen. After grinding the tissue with a mortar and a pestle, the powder was allowed to thaw in 2 ml protein extraction buffer per gram of tissue (25 mM Tris pH 8, 1 mM EDTA, 150 mM NaCl, 5 mM DTT, 0.1% NP-40, 1 Roche complete protease inhibitor cocktail^2^ and 2% PVPP). Extracts were centrifuged at 12,000 *g*, 4°C for 10 min, and the supernatant was passed over four layers of Miracloth^3^ to obtain a “total” protein lysate. 20 μl samples were mixed with Laemmli sample buffer and were run on 13% SDS-PAGE gels and blotted on PVDF membranes using semi-dry blotting. Skimmed milk powder (5%) was used as a blocking agent. The membranes were subjected to immunoblotting using anti-Avr2 antibody (1:10,000 diluted) (Ma et al., 2015). The secondary antibody goat-anti-rabbit (P31470, Pierce) was used at a 1:5000 dilution. The luminescent signal was visualized by ECL using BioMax MR film ^4^.

### Isolation of Apoplastic Fluid from Tomato Leaf Tissue

Apoplastic fluid of tomato plants was isolated as described (Joosten, 2012). Four-week-old fully stretched tomato leaves or leaflets were harvested and placed in a beaker with sterile water. The beaker was placed in a vacuum desiccator and a mild vacuum was employed using a vacuum pump. While slowly releasing the vacuum by opening the vent on the desiccator jar, the leaf tissue became water-soaked and dark in color. The infiltrated leaves were gently dried using tissue papers and then rolled up and placed in a 20 ml syringe hanging in a 50 ml tube. Apoplastic fluid was isolated by centrifuging at 1,000 *g* for 10 min at 4°C. For electrophoresis 20 μl of collected apoplastic fluid was mixed with Laemmli sample buffer and separated on a 13% sodium dodecyl sulfate (SDS) polyacrylamide gel.

### Xylem Sap Collection from Tomato

Xylem sap was collected as described (Rep et al., 2002; Krasikov et al., 2011). Briefly, stems of 6-week-old tomato plants were cut below the second true leaf and the plant was placed in a horizontal position. Then, for minimal 6 h sap bleeding from the cut surface was collected in tubes placed on ice. For electrophoresis 20 μl of collected xylem sap was mixed with Laemmli sample buffer and after heating separated on a 13% SDS polyacrylamide gel.

### *Fusarium* Inoculation Assay

*Fol* was grown in minimal medium (100 mM KNO_3_, 3% sucrose and 0.17% Yeast Nitrogen Base without amino acids or ammonia) and spores were harvested after 3–5 days of cultivation at 25°C with shaking. After washing with sterilized water the spores were diluted to 10^7^ spore/ml. For bioassay, 10-day-old tomato seedlings were uprooted from the soil. The seedlings were placed for 5 min in the *Fol* spore suspension (10^7^ spores/ml) and potted. Disease progression was evaluated after 3 weeks. Plant weight and disease index (Gawehns et al., 2014) were scored for 20 plants/treatment. Using PRISM 5.0 (GraphPad^5^) a pairwise comparison for plant weight was done using the Student’s *t*-test and disease index data was analyzed using a non-parametrical Mann–Whitney *U*-test.

### *Agrobacterium*-Mediated Transient Transformation in Tomato Leaves

The binary ctapi::*GUS* and ctapi::*ΔspAvr2* constructs (Houterman et al., 2009) were transformed into *A. tumefaciens* 1D1249 (Wroblewski et al., 2005). *Agrobacterium*-mediated transient transformation was performed as described (Ma et al., 2012). Briefly, the agrobacteria were grown to an absorbance of 0.8 at 600 nm in LB-mannitol medium (10 g/l tryptone, 5 g/l yeast extract, 2.5 g/l NaCl, 10 g/l mannitol) supplemented with 20 μM acetosyringone and 10 mM MES pH 5.6. Cells were pelleted by centrifugation at 4000 *g* at 20°C for 10 min and then suspended in infiltration medium at an absorbance of 0.5. (1× MS salts, 10 mM MES pH 5.6, 2% sucrose, 200 μM acetosyringone). Infiltration was done in 4-week-old tomato leaves.

### Trypan Blue Staining

Leaves were boiled for 5 min in a 1:1 mixture of 96% ethanol and staining solution (100 ml lactic acid, 100 ml phenol, 100 ml glycerol, 100 ml H_2_O, and 100 mg Trypan bule). The leaves were destained in 2.5 g/ml chloral hydrate in water (Ma et al., 2012).

### *V. dahliae* Inoculation Assay

Ten-day-old tomato plants were carefully uprooted from the soil and the roots were placed in a race 1 *Verticillium dahliae* JR2 inoculum (1 × 10^6^ conidia/ml) for 5 min (Fradin et al., 2009). Thereafter, the plants were transferred to fresh soil. After 2 weeks disease symptom were scored by measuring the canopy surface and fresh weight of the plants (Fradin et al., 2009). A one-way ANOVA with Dunnet’s *post hoc* test for weight and leaf area was performed using PRISM 5.0. Fungal colonization in tomato plants was assessed at 21 dpi. Stem sections at the position of the cotyledon, second node and fourth node were collected separately. The stem pieces were surface-sterilized in 70% ethanol, rinsed in sterile distilled water, and the ends of the stem that had been exposed to the water were removed with a sterile scalpel. Stem sections of about 5 mm thick were cut and placed on potato dextrose agar (PDA) plates supplemented with 200 mg/l streptomycine and 100 mg/l penicillin at 25°C, allowing the fungus to grow out of the stem sections. Pictures were taken after 5 days of incubation at 25°C. Data are expressed as a percentage of infected slices.

### Grafting

Four-week-old rootstocks and scions represent the best stage for grafting ^6^. A similar diameter of the stem of the rootstock and scion increases the likelihood that their vasculatures align after grafting. The rootstock plant was cut between the cotyledons and first true leaf. The scion plant was cut at the same position at the main stem. Leaves from the scion were trimmed to reduce water loss. The stump of the scion seedling was cut to fit the shape of a two-sided wedge. Approximately, one-third of each side was removed at a roughly 45° angle. The stump of the scion seedling was trimmed on both sides, creating a wedge with angled sides of approximately 45°. The wedge-shaped scion stump was inserted into the cut of the bisected rootstock stump. Parafilm was used to fix the rootstock and scions and to secure the graft. Grafted plants were placed for 5 days in a growth chamber with high humidity to reduce dehydration stress and increase the survival rate.

## Results

### Avr2 Exerts Its Virulence Function Inside Host Cells

**FIGURE 1:**
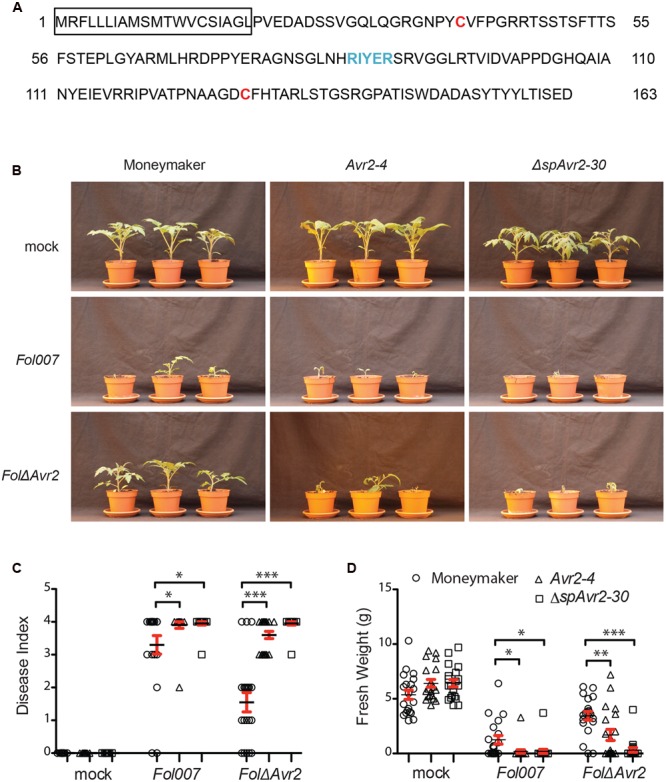
**Avr2 exerts its virulence function inside host cells. (A)** A schematic diagram of Avr2 in which the signal peptide is boxed and the two cysteine residues and the predicted RxLR (Arg-x-Leu-Arg)-like motif are marked red and blue, respectively. **(B)** Ten-day-old seedlings of wild-type (Moneymaker), full-length *Avr2-4* and *ΔspAvr2-30* transgenic tomato plants were inoculated with water (mock), wild-type *Fusarium Fol007* or *FolΔAvr2*. Three weeks after inoculation, **(C)** average disease index and **(D)** mean plant weight of 20 plants were scored. Error bar represent mean ± SE (^∗^*P* < 0.05; ^∗∗^*P* < 0.01; ^∗∗∗^*P* < 0.001). For clarity only one representative transgenic line is shown.

Avr2 was originally identified in the xylem sap of *Fol*-infected tomato plants (Houterman et al., 2007), although a nuclear localization of Avr2 is required to trigger I-2-mediated resistance (Ma et al., 2013). As yet it is unknown where in the host the protein exerts it virulence function. To identify whether Avr2 acts inside or outside host cells, transgenic tomato plants stably expressing full-length *Avr2* were generated. The expressed protein carries it’s endogenous signal peptide (**Figure 1A**) for translocation into the endoplasmatic reticulum and subsequent secretion. Plant-produced Avr2 is, therefore expected to be secreted into the apoplastic spaces, allowing us to test whether it exerts its function extracellularly. In addition, stable transgenic plants were made expressing a truncated *Avr2* (*ΔspAvr2*,Δsp for *“*deletion of signal peptide”) encoding the mature protein without signal peptide (Ma et al., 2013). In these plants the protein is predicted to be present exclusively in the cytosol. Expression of both full-length *Avr2* and *ΔspAvr2* was driven by the strong and constitutive CaMV 35S promoter. Both genes were fused to sequences encoding a C-terminal HA and SBP tag to facilitate detection of the recombinant proteins.

From kanamycin resistant T1 plants three independent transformants containing a single copy of the *Avr2* construct (*35S::Avr2-1, 35S::Avr2-4*, and *35S::Avr2-7*) and two lines containing a single copy *ΔspAvr2* lines (*35S::ΔspAvr2-3* and *35S::ΔspAvr2-30*) were identified based on their 3:1 segregation pattern. From these plants homozygous lines were produced, which were used in the subsequent assays. None of the transgenic lines exhibited morphological aberrations, or showed a phenotype distinct from non-transformed Moneymaker tomato plants when grown under standard greenhouse conditions. To determine whether the distinctively localized Avr2 effector proteins do complement the virulence defect of a *FolΔAvr2* (a *Fol Avr2* knockout; Houterman et al., 2009) strain, 10-day-old seedlings of wild-type, *ΔspAvr2* and full-length *Avr2* transgenic tomato plants were inoculated with water (mock), wild-type *Fusarium* (*Fol007)* or the *FolΔAvr2* strain. Three weeks after inoculation, mean plant weight and average disease index of 20 plants was scored. The disease index was scored on a 0–4 scale, in which 0 means that no disease symptoms developed and 4 that plants are either dead or extremely small and wilted (Gawehns et al., 2014). Moneymaker plants inoculated with *Fol007* showed severe disease symptoms such as wilting and stunting (**Figure 1B** shows a representative example of lines *ΔspAvr2-30* and *Avr2-4*). As observed before (Houterman et al., 2007), the *FolΔAvr2* strain is reduced in virulence as shown by the increased vigor of the plants along with higher weights and lower disease indexes as compared to *Fol007* inoculation (**Figures 1C,D**). Interestingly, we found that disease symptoms of *FolΔAvr2-*infected *Avr2* plants were at least as severe as tomato plants infected with wild-type *Fol*. The regain of full pathogenicity for the *Fol Avr2* knockout strain on the *Avr2* lines shows that plant-produced Avr2 effectively complements fungal virulence. Notably, also *ΔspAvr2* tomato plants infected with *FolΔAvr2* developed severe disease symptom and showed decreased plant weight and a higher disease index. Since the latter protein does not carry a signal peptide, this strongly suggests that Avr2 exerts its virulence functions inside the host cell. The experiment was performed twice using all five transgenic lines, with similar results.

### In *Avr2* Transgenic Plants Avr2 is Secreted into the Xylem Sap and the Apoplast

**FIGURE 2:**
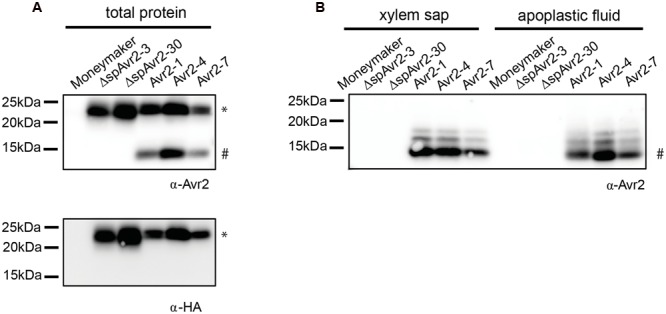
**Avr2 accumulates in xylem sap and apoplastic fluids of *Avr2* plants. (A)** Western blot showing accumulation of HASBP-tagged Avr2 in total protein extracts of transgenic tomato plants expressing either full-length *Avr2* or *ΔspAvr2*. The top blot was probed with an antibody targeted against Avr2 while the bottom blot was developed using an HA antibody. The top band (^∗^) corresponds to the size of HASBP-tagged Avr2 whereas the lower band (#) represents the size of a non-tagged Avr2. **(B)** Western blot of xylem sap and apoplastic fluid isolated from the above mentioned plants probed with an Avr2 specific antibody. Avr2 accumulates in apoplastic fluid and xylem sap of transgenic tomato plants expressing full-length *Avr2-*, but not in plants expressing*ΔspAvr2.* The molecular weight, as indicated by the precision plus protein standard (Bio-Rad), is shown on the left.

To assess accumulation of Avr2 in the transgenic tomato plants, the *35S::Avr2* and *35S::ΔspAvr2* lines were subjected to immunoblot analysis using either an Avr2 specific antibody (Ma et al., 2015) or an HA antibody (**Figure 2A**). When probed with Avr2-specific antibody, a band with the predicted size for ΔspAvr2-HASBP (23 kDa) was detected in total protein extracts from *Avr2* and *ΔspAvr2* transformants, but not in the untransformed Moneymaker control plants confirming the specificity of the antibody. In the *Avr2* transformants also one additional band of a smaller size (15 kDa) was observed. The appearance of this smaller sized band suggests that Avr2 is secreted into the apoplastic spaces, after which the HA tag is cleaved by extracellular proteases (van Esse et al., 2006). The apparent weight fits the predicted size of the non-tagged Avr2 protein. When probed with HA antibody only the larger 23 kDa band was detected, which indicates that the 15 kDa band indeed contains the non-tagged ΔspAvr2 protein from which the tag has been removed. To determine the *in planta* location of the ΔspAvr2 and full-length Avr2 proteins apoplastic fluid and xylem sap were isolated from the transgenic plants. Western blot analysis of these fluids revealed that the 15 kDA Avr2 protein is present in the apoplastic fluid and xylem sap of *Avr2* plants (**Figure 2B**). Its presence in the sap shows that (i) the 35S promoter is active in the mesophyll and xylem-adjacent cells and (ii) the signal peptide of *Fol* is functional and (iii) the protein is secreted. No Avr2 protein was detected in the extracellular fluids of *ΔspAvr2* plants, which shows that Avr2 is not secreted and hence must fulfill its virulence function inside the cell. Therefore, complementation of the compromised virulence of the *FolΔAvr2* strain in *Avr2* plants is either due to re-uptake of secreted Avr2, or due to the activity of a cytoplasmic Avr2 pool that evaded signal peptide-mediated secretion from the plant cell.

### *I-2-*Expressing Xylem-Adjacent Cells Do Not Take Up Avr2 Host-Autonomously

**FIGURE 3:**
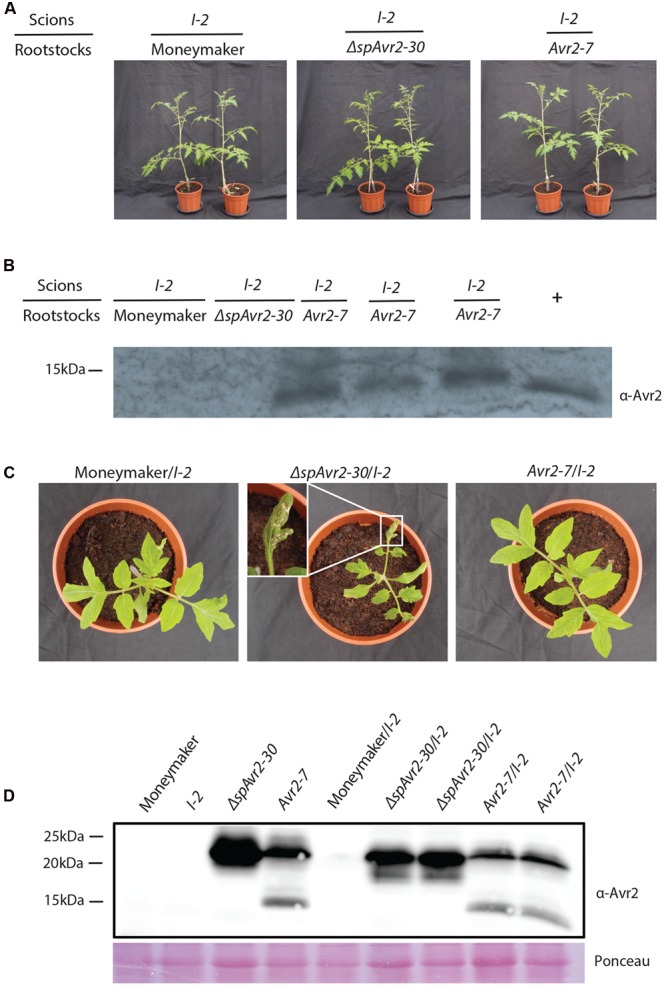
***I-2-*carrying tomato plants do not trigger immune signaling upon Avr2 exposure. (A)** Scions of 4-week-old tomato plants expressing *I-2* grafted onto a wild-type Moneymaker, a *ΔspAvr2* or an *Avr2* rootstock. Representative grafts are shown 4-weeks-post grafting. Note that all grafts grew normally and did not develop autoimmune symptoms **(B)** Western blot analysis of xylem sap harvested ±10 cm above the graft. The Avr2 protein could be readily detected in xylem sap of *I-2* scions placed on an *Avr2* rootstock, but not in xylem sap isolated from scions grafted on either wild-type or a *ΔspAvr2* roots stock. As a positive reference Avr2-containing xylem sap was harvested from tomato plants inoculated with *Fol007*. **(C)** *Avr2-7* and *ΔspAvr2-30* transformants were crossed to *I-2* tomato plants. Two weeks after germination *ΔspAvr2/I-2* plants developed clear autoimmune phenotypes; i.e., necrotic lesions, reduced plant weight and stunted growth, whereas no symptoms developed on Moneymaker*/I-2* or *Avr2/I-2* progeny. **(D)** Western blot analysis shows accumulation of Avr2 in *Avr2* and *ΔspAvr2* transgenic tomato plants and their *ΔspAvr2/I-2* and *Avr2/I-2* progenies. The blot was probed with an antibody targeted against Avr2. Lower panel shows a Ponceau S staining that serves as loading control.

To determine whether tomato cells can take up Avr2 via a host-autonomous process, grafting studies were performed in which scions of tomato plants expressing *I-2* were grafted onto wild-type Moneymaker, *ΔspAvr2* or *Avr2* rootstocks. Since Avr2 is present in the xylem sap of *Avr2* plants, through which water and nutrients are transported from roots to shoot and leaves, the effector is predicted to be transported from the *Avr2* rootstock into the *I-2* scion. If the *I-2-*expressing cells autonomously take up the effector protein from the xylem sap then I-2-mediated immune responses will be induced. As predicted, and shown in **Figure 3A**, no difference in growth was observed when an *I-2* scion was placed on either a wild-type or a *ΔspAvr2* rootstock. The lack of growth retardation or necrosis in the chimeras grafted on a *ΔspAvr2* rootstock is consistent with the observation (**Figure 3A**) that ΔspAvr2 is not secreted and hence cannot be translocated through the plant to *I-2* expressing tissues. Notably, also no I-2 immune symptoms appeared in *I-2* scions grafted on an *Avr2* rootstock (**Figure 3A**). Per genotype combination at least ten independent grafts were made and in none of them an autoimmune response was observed. The lack of I-2 activation suggests that either Avr2 is not transported to the upper part of the plant, or that it is not taken up from the xylem sap. To distinguish between these options Western blot analysis on xylem sap was done. Xylem sap was harvested from stems cut at ±10 cm above the graft to exclude possible contamination of the sap with Avr2 leaking out of damaged *Avr2* expressing cells. As can been seen in **Figure 3B** the Avr2 protein could readily be detected in the xylem sap of *I-2* scions placed on an *Avr2* rootstock, but not in xylem sap isolated from scions grafted on either wild-type of a *ΔspAvr2* root stocks.

These results show that Avr2 is transported from the *Avr2* rootstock into *I-2* scions and that the absence of I-2-mediated immune elicitation is either due to inability of the plant to autonomously take up Avr2 from the xylem sap, or that the effector concentration is too low to trigger an I-2-mediated response. To examine both options non-transgenic Moneymaker and *ΔspAvr2-30* and *Avr2-7* transgenic tomato plants were crossed with *I-2* tomato plants. Combining the resistance and avirulence gene into one plant ensures a systemic presence of both proteins and a high effector abundance inside the plant. From all three crosses F1 seeds were obtained and 15 seeds per cross were analyzed for their ability to germinate. No differences in germination frequency or timing were observed. The seedlings of the different progeny were indistinguishable from each other during the first 2 weeks following germination. However, whereas Moneymaker/*I-2* plantlets continued to grow normally and developed into mature plants bearing fruits, the *ΔspAvr2/I-2* progeny developed a clear auto-immune phenotype; necrotic lesions emerged on the leaves and the plants showed reduced weight and stunted growth (**Figure 3C**). Although the *ΔspAvr2/I-2* plants continued to grow and even flowered, they never developed fruits. The autoimmune phenotype of the *ΔspAvr2/I-2* plant is consistent with the intracellular recognition of the protein by the I-2 immune receptor (Ma et al., 2013) and confirms that tomato leaf cells are capable of showing an I-2 response upon exposure to cytoplasmically localized Avr2. Therefore, it was interesting to observe that no necrotic lesions developed on the *Avr2/I-2* progeny. The lack of I-2 activation in these plants implies that secreted Avr2 is not taken up from the extracellular spaces, either the xylem or apoplast, to trigger an I-2 response. The presence of the *Avr2* and *I-2* genes in *ΔspAvr2/I-2* and *Avr2/I-2* progenies was verified by PCR using *Avr2* and *I-2* specific primers on genomic DNA (**Supplementary Figure S1A**). Additionally, Western blot analysis of the leaves of parental lines and their F1 progeny (*ΔspAvr2/I-2* and *Avr2/I-2*) revealed that Avr2 is not only present in the *ΔspAvr2* and *Avr2* transgenic parental plants but also in their *ΔspAvr2/I-2* and *Avr2/I-2* progeny (**Figure 3D**; **Supplementary Figure S1B**). Taken all together, the lack of Avr2-mediated I-2 activation in *Avr2/I-2* plants suggests that *I-2-*expressing cells cannot autonomously take up the Avr2 effector protein from the extracellular spaces.

### Infiltration of *Agrobacterium* in *Avr2/I-2* Tomato Leaves Triggers HR

**FIGURE 4:**
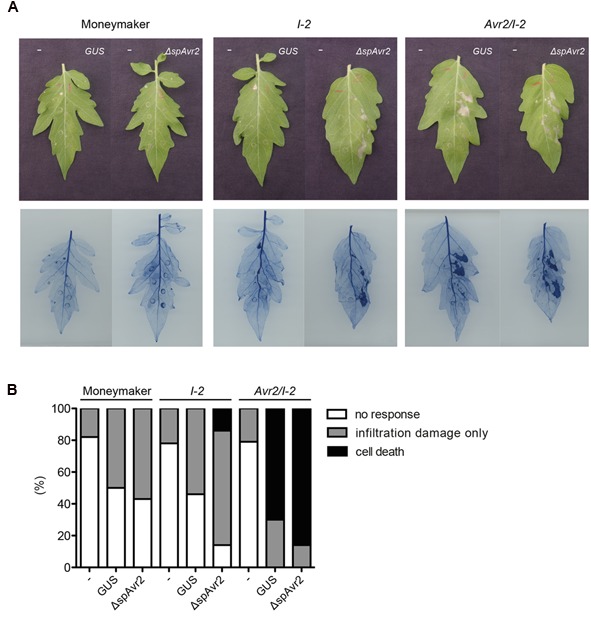
**Infiltration of *Agrobacterium tumefaciens* in *Avr2/I-2* tomato plants triggers cell death. (A)** Four-week-old wild-type Moneymaker, *I-2* and *Avr2/I-2* tomato plants infiltrated with either infiltration buffer (“-”) or *Agrobacterium* expressing *GUS* or *ΔspAvr2*. The left side of each leaf is buffer infiltrated and the right site is infiltrated with *Agrobacterium* carrying either a *GUS* or *ΔspAvr2* construct. Photographs were taken 4 dpi. The bottom panel shows the same leaves stained with trypan blue to visualize cell death. **(B)** 20 leaves of wild-type Moneymaker, *I-2* and *Avr2/I-2* tomato plants were scored for their response following infiltration. The assay was repeated twice with similar results.

Previously it was reported that agro-infiltration of either an *Avr2*- or a *ΔspAvr2*-encoding construct triggers *I-2*-dependent HR in *Nicotiana benthamiana* (Houterman et al., 2009). Since the signal peptide of Avr2 is functional *in planta* (**Figure 2**), this finding suggests uptake of the secreted Avr2 protein by the plant cells in the presence of *A. tumefaciens.* The *Avr2/I-2* plants allow us to test this hypothesis. The expectation is that upon *A. tumefaciens* infiltration cell death will be triggered in the transgenic plants, but not in wild-type tomato. The A. *tumefaciens* 1D1249 strain was used to infiltrate tomato as, unlike most laboratory strains, it does not trigger necrosis in the leaf (Wroblewski et al., 2005). Four-week-old wild-type Moneymaker, *I-2* and *Avr2/I-2* tomato plants were infiltrated with *A. tumefaciens* 1D1249 delivering either *GUS*, which serves as a negative control, or *ΔspAvr2* acting as positive control for I-2 mediated cell death. To be able to distinguish specific responses from non-specific ones also a mock infiltration was done using buffer without *A. tumefaciens*. To better visualize the occurrence of cell death the leaves were stained with trypan blue. At 4 days post infiltration (dpi) the majority (80%) of mock infiltrated leaves were symptomless, although some cell death directly beneath the infiltration sites was found (**Figures 4A,B**). Infiltration of *Agrobacterium* delivering either *GUS* or *ΔspAvr2* in wild-type plants also only showed cell death at the infiltration sites itself, and not in the sector around it, which can be attributed to mechanic damage. In contrast to this, agro-infiltration of *ΔspAvr2*, but not *GUS*, triggered cell death in a sector around the infiltration points in 20% of the infiltrated *I-2* tomato leaves. The induction of *I-2*-dependent cell death following transient expression of *ΔspAvr2* is consistent with the former observations in *N. benthamiana*, and shows that 1D1294 can be used for transient transformation of tomato and that tomato leaves are capable of mounting an *I-2* specific response upon Avr2 perception.

In contrast to the *I-2* plants, which only responded to an *A. tumefaciens* strain carrying *ΔspAvr2*, the majority of *Avr2/I-2* leaves exhibited a strong cell death response of the infiltrated sector following agro-infiltration of either strain. In respectively 70 and 80% of the *Avr2/I-2* leaves cell death was induced after infiltration of either *GUS* or the *ΔspAvr2* construct. The cell death is independent of the construct, but requires *A. tumefaciens* as necrosis was not induced in the mock infiltrated sectors. Together these data shows that *A. tumefaciens* infiltration triggers cell death in *Avr2/I-2* tomato plants, likely by facilitating the uptake of Avr2 by the plant cells.

### *Verticillium dahliae* Does Not Facilitate Avr2 Uptake by Tomato Cells from the Extracellular Space to Exert its Intracellular Virulence Function

In **Figure 1** it is shown that both *ΔspAvr2* and *Avr2* transgenic tomato lines fully complement the virulence defect of a *Fol ΔAvr2* knockout, implying that *Fol* facilitates Avr2 uptake from the extracellular spaces into plant cells. To test whether effector uptake is a generic phenomenon that can be induced by any vascular fungal pathogen, we tested whether *V. dahliae* can also induce effector uptake. If Avr2 targets host processes that are important for pathogenicity of *V. dahliae* then it is predicted that *ΔspAvr2* plants show hyper-susceptibility to the fungus. If so, the degree of disease susceptibility of the *Avr2* plants could be used as a proxy to monitor effector uptake.

**FIGURE 5:**
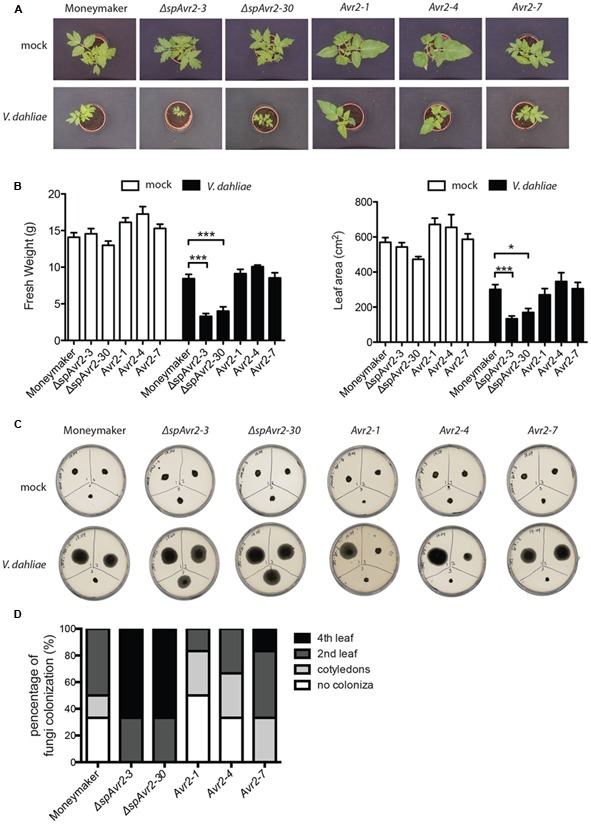
***Verticillium dahliae* does not facilitate Avr2 uptake in tomato. (A)** Representative pictures of mock (upper row) and *V. dahliae* race 1 JR2 (bottom row) inoculated Moneymaker, *ΔspAvr2* and *Avr2* transgenic tomato plants at 21 days post inoculation (dpi). Hyper-susceptibility was only observed in *ΔspAvr2* plants. As a measure for disease development **(B)** fresh weight and leaf canopy surface of inoculated plants was measured. Error bar represent mean ± SE. (^∗^*P* < 0.05; ^∗∗∗^*P* < 0.001). **(C)** As a measure of *V. dahliae* colonization stem sections from the cotyledon (top-left), second node (top-right) and fourth node (bottom-center) of each plant were collected at 21 dpi and placed on PDA plates. Pictures were taken 5 days post incubation on plate. **(D)** Fungal progression in the stem was expressed as a percentage of infected slices.

Besides wild-type Moneymaker, two independent *ΔspAvr2*-expressing tomato lines (*ΔspAvr2-3* and *ΔspAvr2-30*) and three independent *Avr2* tomato lines (*Avr2-1, Avr2-4*, and *Avr2-7*) were used in the *V. dahliae* bioassay. Stunting, chlorosis, necrosis and vascular browning are typical symptoms of *Verticillium* wilt disease. Hence disease symptoms following inoculation can be quantified by measuring the canopy surface and fresh weight of inoculated plants. *V. dahliae*-inoculated wild-type Moneymaker plants showed moderate stunting, as compared to mock-inoculated plants, confirming successful infection (**Figure 5A**). Interestingly, *V. dahliae-*inoculated *ΔspAvr2* plants were much smaller in stature and showed a significant reduction in canopy surface and fresh weight as compared to the inoculated Moneymaker plants (**Figure 5B**). The enhanced disease symptoms and hyper-susceptibility of the *ΔspAvr2* plant implies that cytoplasmatically localized Avr2 enhances the virulence of *V. dahliae* allowing us to use disease development as a proxy for effector uptake. It was, therefore, interesting to note that *V. dahliae* infected *Avr2* plants did not show hyper-susceptibility as the symptoms on these plants were indistinguishable from the non-transgenic controls. The bioassay was repeated three times with similar results (data not shown). The lack of hyper-susceptibility of *Avr2* tomato plants suggests that extracellular Avr2 is not taken up by *V. dahliae* infected plant cells in quantities sufficient to reveal its intracellular virulence-promoting activity.

To investigate in further detail whether the hyper-susceptibility in *ΔspAvr2* plants correlates with increased fungal colonization, a fungal recovery assay was performed (Fradin et al., 2009). To this end, stem sections were taken from *V. dahliae* inoculated tomato plants at positions of (1) the cotyledon, (2) the second node, and (3) the fourth node, and these were placed on PDA plates and incubated for 5 days at 25°C. The fungus could only be recovered from lower stem sections of Moneymaker and *Avr2* plants whereas *Verticillium* grew out from all stem sections of *ΔspAvr2* plants (**Figure 5C**). *Verticillium* colonized over 60% of *ΔspAvr2* plants till the fourth node, whereas it could typically only reach the cotyledon level or second node of wild-type Moneymaker and *Avr2* plants (**Figure 5D**). Overall, these data show that the *ΔspAvr2*, but not *Avr2* plants are hyper-susceptible toward *V. dahliae* as depicted by their enhanced fungal colonization and increased disease symptoms. These data suggest that *V. dahliae* does not facilitate Avr2 uptake by tomato cells from the extracellular spaces to exert its intracellular virulence functions.

## Discussion

Here, we show that expression of either full length *Avr2* or *ΔspAvr2* in tomato complements the compromised virulence of a *FolΔAvr2* strain. Hence, Avr2 not only exerts its avirulence function intracellularly (Ma et al., 2013), but also its virulence activity. How the protein exerts its virulence function is unknown, but apparently it targets a process that is also important for infection of *V. dahliae* as *ΔspAvr2* plants became hyper-susceptible to this fungus. The observation that extracellular plant-produced Avr2 fully complements the *FolΔAvr2* strain implies that the protein is either able to evade signal peptide-mediated secretion or is taken up by the plant cells. Since, we detected Avr2 in xylem sap and apoplastic fluid of *Avr2* transgenic plants the signal peptide must be functional lending support to the second hypothesis. In agreement, it has been reported that Avr2 is secreted into tomato xylem vessels by *Fol* (Houterman et al., 2007), while it intracellularly activates *I-2* (Houterman et al., 2009; Ma et al., 2013) further indicating that Avr2 is taken up by plant cells in the presence of *Fol*.

How host cells take up effectors is unknown and monitoring effector movement from the pathogen to the host cell is technically challenging (Petre and Kamoun, 2014). Also our attempts to directly visualize uptake using *Fol* strains producing GFP tagged effector proteins were unsuccessful as the tags were cleaved off by extracellular proteases, like they are in this study (**Figure 2A**). Mostly two types of assays are used to monitor the ability of effector proteins to enter host cells; “the cell re-entry” and the “protein uptake” method (Catanzariti et al., 2006; Dou et al., 2008a; Kale and Tyler, 2011). However, both assays have their limitations (Petre and Kamoun, 2014; Lo Presti et al., 2015). The first has the drawback that it is unclear whether effectors had indeed been secreted into the apoplast prior to re-internalization, and it is therefore not possible to exclude that effectors might have escaped the secretory pathway and thus remained in the cytoplasm (Oh et al., 2009). In the second assay protein is infiltrated in leaves, or added to cell suspension in which cells are stressed or wounded, which might trigger non-specific protein internalization complicating the interpretation of the data (Yaeno et al., 2011; Wawra et al., 2013).

We have overcome the limitation of these former assays by using an unique functional assay in which effector production is spatially separated from its action, and in which no wounding is involved. Our grafting experiment shows that although Avr2 is present in the xylem sap of the *I-2* graft, and hence is translocated from the *Avr2* rootstock, it is unable to trigger *I-2*-mediated immune responses in the *I-2* scion. These data suggest that *I-2-*expressing cells do not autonomously take up the effector protein from the xylem sap in the absence of *Fol*. Crosses between either *Avr2* or *ΔspAvr2* plants with *I-2* tomato substantiate this conclusion, as an autoimmune response was only observed in the *ΔspAvr2/I-2* crosses. Furthermore, the lack of an immune response in the *Avr2/I-2* plants implies that secretion of Avr2 is a very efficient process. So although Avr2 is abundantly present in the extracellular spaces of *Avr2/I-2* progeny, the secreted Avr2 is not perceived by intracellular I-2 again implying that plant cells do not autonomously take up the Avr2 effector.

Notably, in the presence of *Fol* the secreted Avr2 protein is able to enter the host cell as it complements the virulence defect of a *FolΔAvr2* strain. This observation implies that during infection a factor is produced that is required for Avr2 uptake by the host cell. The identity of this factor is unknown, but since infiltration of *A. tumefaciens* also stimulated effector uptake, the property to generate this a factor seems to be shared by other plant pathogens. Agro-infiltration of either an *Avr2*- or a *ΔspAvr2*-encoding construct was previously shown to trigger *I-2*-dependent HR in *N. benthamiana*, suggesting uptake of secreted Avr2 in the presence of the bacterium (Houterman et al., 2009). In line with this finding, we here show that agroinfiltration of *Avr2/I-2* leaves triggers cell death irrespectively of the construct carried by the bacterium. The ID1294 strain containing the *ΔspAvr2*-encoding construct triggers a relative weak cell death response in *I-2* tomato, which is in line with the reported low transient transformation efficiency of this strain (Wroblewski et al., 2005). In agroinfiltrated leaves of *Avr2/I-2* plants slightly more cell death was induced by the *ΔspAvr2* carrying *A. tumefaciens* strain than by the *GUS* control strain. This difference is likely attributable to a higher cytosolic Avr2 concentration due simultaneous uptake and production of Avr2 in the *Avr2/I-2* cells. Nevertheless, it is clear that the mere presence of the bacterium in *Avr2/I-2* plants is sufficient to trigger cell death. These findings are especially relevant for studies in which pathogen-independent uptake was suggested based on assays in which the effectors were expressed using *A. tumefaciens* (Rafiqi et al., 2010; Kloppholz et al., 2011).

Given that *A. tumefaciens* has the property to trigger effector uptake, it was surprising to find that *Avr2-*expressing plants did not become hyper-susceptible to *V. dahliae*, as it suggests that this pathogen does not induce effector-uptake by host cells. That Avr2 can confer hyper-susceptibility was shown by the *ΔspAvr2* plants; compared to control plants the *ΔspAvr2* plants show a significant reduction in canopy surface and fresh weight following *V. dahliae* infection. Together these results imply that *V. dahliae* either does not produce the factor that is required for effector uptake, or that the fungus does not produce it in sufficient amounts to exert a measurable effect in the bioassay. The latter would be in line with the very low amount of fungal biomass typically observed in *V. dahliae* infected plants (Faino et al., 2012). Future experiments, using other plant pathogenic fungi and bacteria can reveal whether the ability of plant pathogens to trigger effector uptake depends on the type of pathogen, or just on the amount of microbial biomass, or both. The materials described in this paper are perfectly suited to address this question.

Uptake of effectors by plants has been described before (Dou et al., 2008b; Kale et al., 2010), but it is currently unclear which properties determine whether an effector can be taken up by the host. For the AvrM and AvrL567 effectors from the flax rust fungus *Melampsora lini* it was shown that their N-termini are required for translocation into host cells when transiently expressed using *A. tumefaciens* transformation (Ve et al., 2013). For ToxA from *Pyrenophora tritici-repentis* the C-terminal RGD motif is involved in internalization (Manning et al., 2008). The Avr2 protein does not show sequence homology with the flax rust effector proteins, nor does it contain a clearly distinguishable RGD motif. However, it has been proposed that Avr2 contains an RxLR (Arg-x-Leu-Arg)-like motif (**Figure 1A**) that might be involved in its uptake (Kale et al., 2010). The RxLR and DEER motifs (Asp-Glu-Glu-Arg) are frequently found in oomycete effectors (Bhattacharjee et al., 2006; Tyler et al., 2006; Jiang et al., 2008) and have been shown to function as a host-targeting signal allowing the protein to be translocated into host cells (Whisson et al., 2007; Dou et al., 2008a). Kale et al. (2010) reported that the RxLR motif allows effectors to bind phosphatidylinositol-3-phosphate (PI3P) present on the outer surface of the plant plasma membrane enabling vesicle-mediated endocytosis. However, whether the RxLR-like motif in Avr2 is functional and required for uptake remains unclear as mutations in the motif rendered the protein unstable when transiently expressed in the plant prohibiting functional analysis (Ma, 2012).

Samuel et al. (2015) proposed that effectors could be transferred by extracellular vesicles (EVs). Proteins lacking secretion signals could be packaged into EVs for passage through the plasma membrane whilst proteins containing a secretion signal could be secreted into the matrix of the cell wall and then bind to EVs via a lipid binding motif. The protein then transits the cell wall as a passenger on the outer leaflet of the vesicle (Samuel et al., 2015). Whether such a mechanism applies to Avr2 is unclear. An indication that the effector might associate with vesicles is the observation that RFP-tagged Avr2 expressed from *Fol* during colonization of the xylem vessel forms red-fluorescent punctate spots alongside the mycelium where it touches the plant cells (Ma, 2012). A local high concentration of the RFP-labeled effector is consistent with the protein being sequestered on a specific location from where it could be internalized into vesicles. How an effector protein subsequently dissociates from a vesicle and is released in the host cytoplasm remains unclear; possibly the unknown factor produced during infection plays a determining role in this process. The nature of the factor triggering effector uptake is unknown. It might be another secreted effector protein from the pathogen, a microbial metabolite or a plant signal generated upon pathogen-inflicted damage. Since both *A. tumefaciens* and *Fol* trigger effector uptake we favor the latter option, also because the “protein uptake” method shows effector uptake by *in vitro* grown cultured plant cells in the absence of a microbe (Dou et al., 2008a; Kale and Tyler, 2011). Future studies are required to reveal the identity of this elusive factor.

## Conclusion

We here describe a series of functional assays demonstrating that tomato cells do not take up the *Fol* Avr2 effector protein in the absence of a plant pathogen. Effector uptake was shown in the presence of both *Fol* and *A. tumefaciens*. The *Avr2/I-2* tomato plants generated in this study provide an excellent starting point to investigate whether other plant pathogens also have the ability to trigger effector uptake, and to identify the factor responsible for this process.

## Author Contributions

FT and XD planned the experiments, FT supervised the experiments, XD performed most of the experiments, JG performed the *V. dahliae* bioassays, LM created the transgenic *Avr2* tomato lines, FT, XD, and HB designed the experiments and XD and FT analyzed the data. FT conceived the project and wrote the manuscript together with XD. All authors approve the manuscript and are accountable for all aspects of the work.

## Conflict of Interest Statement

The authors declare that the research was conducted in the absence of any commercial or financial relationships that could be construed as a potential conflict of interest.

## Footnotes

1 http://www.mwg-biotech.com

2 http://www.roche.com

3 http://www.merckmillipore.com/NL/en/product/Miracloth,EMD_BIO-475855

4 http://www.sigmaaldrich.com/catalog/substance/carestreamkodakbiomaxmrfilm1234598765?lang=en&region=NL

5 http://www.graphpad.com

6 http://horticulture.ucdavis.edu/main/Deliverables/Kleinhenz/tomato_grafting_guide.pdf

## Supplementary Material

FIGURE S1 **Presence of *Avr2* and *I-2* gene in *ΔspAvr2/I-2* and *Avr2/I-2* tomato plants. (A)** Ethidium bromide stained agarose gel showing the PCR products obtained with either *Avr2* or *I-2* specific primers using DNA extracted from the indicated plants. The GeneRuler 1 kb DNA Ladder (Fermentas) is shown on the left. **(B)** Western blot analysis shows accumulation of Avr2 in the parental *Avr2* and two independent *ΔspAvr2* transgenic tomato plants, and in two independent *ΔspAvr2/I-2* and *Avr2/I-2* progenies. The blot was probed with an antibody targeted against Avr2. The precision plus protein standard (Bio-Rad) is shown on the left.
