# Supplementary figures and images for: Uptake of the Fusarium Effector Avr2 by Tomato Is Not a Cell Autonomous Event
Source: Front Plant Sci. 2016 Dec 21;7:1915. doi: 10.3389/fpls.2016.01915 (PMC5175262; doi:10.3389/fpls.2016.01915)

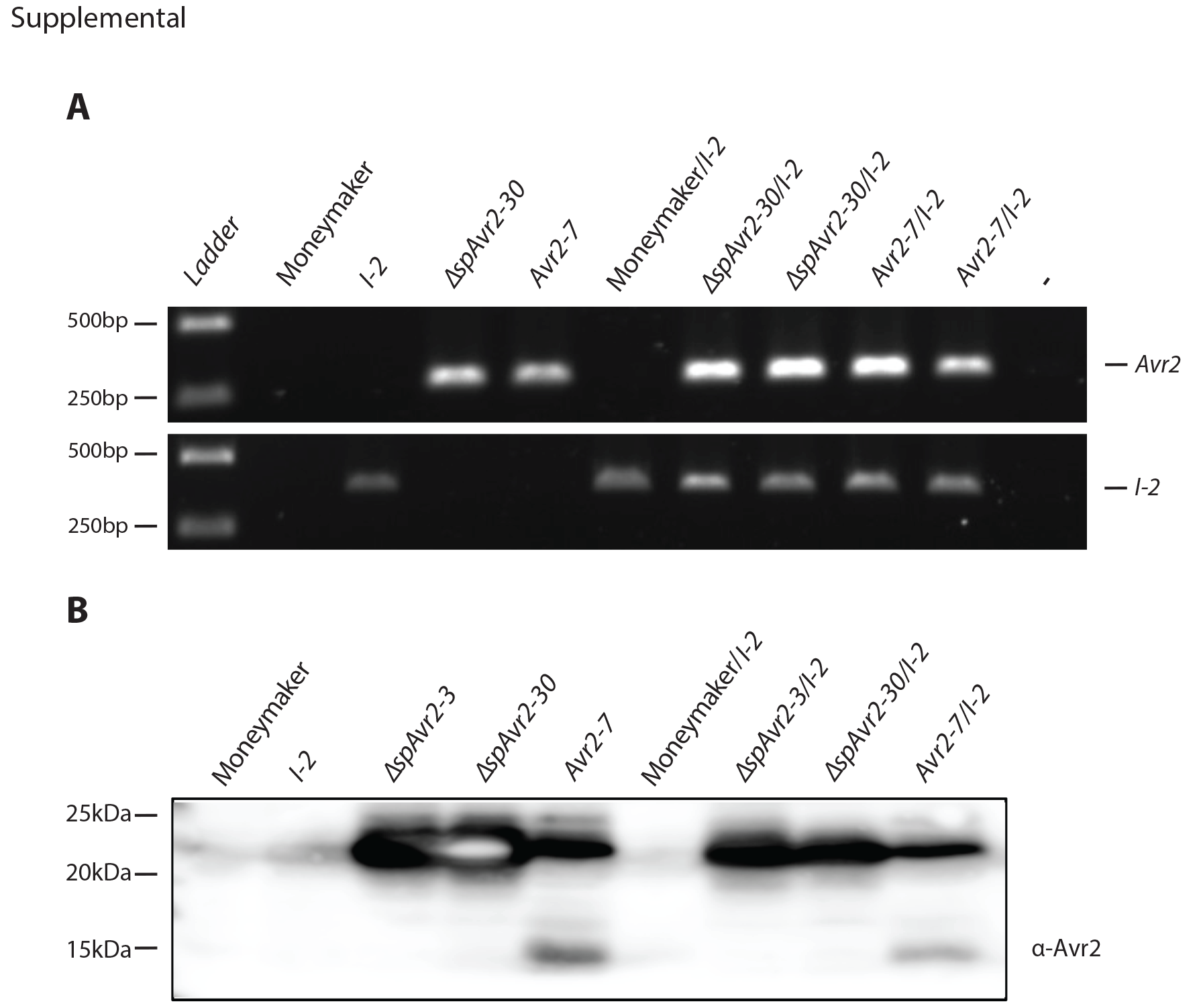

Supplement: FIGURE S1 — Presence of Avr2 and I-2 gene in ΔspAvr2/I-2 and Avr2/I-2 tomato plants. (A) Ethidium bromide stained agarose gel showing the PCR products obtained with either Avr2 or I-2 specific primers using DNA extracted from the indicated plants. The GeneRuler 1 kb DNA Ladder (Fermentas) is shown on the left. (B) Western blot analysis shows accumulation of Avr2 in the parental Avr2 and two independent ΔspAvr2 transgenic tomato plants, and in two independent ΔspAvr2/I-2 and Avr2/I-2 progenies. The blot was probed with an antibody targeted against Avr2. The precision plus protein standard (Bio-Rad) is shown on the left. [file Image_1.TIF]
